# Genome editing of the disease susceptibility gene *CsLOB1* in citrus confers resistance to citrus canker

**DOI:** 10.1111/pbi.12677

**Published:** 2017-01-04

**Authors:** Hongge Jia, Yunzeng Zhang, Vladimir Orbović, Jin Xu, Frank F. White, Jeffrey B. Jones, Nian Wang

**Affiliations:** ^1^ Citrus Research and Education CenterDepartment of Microbiology and Cell ScienceInstitute of Food and Agricultural Sciences (IFAS)University of FloridaLake AlfredFLUSA; ^2^ Citrus Research and Education CenterIFASUniversity of FloridaLake AlfredFLUSA; ^3^ Department of Plant PathologyIFASUniversity of FloridaGainesvilleFLUSA

**Keywords:** *Xanthomonas citri*, Cas9, sgRNA, *Citrus paradisi*

## Abstract

Citrus is a highly valued tree crop worldwide, while, at the same time, citrus production faces many biotic challenges, including bacterial canker and Huanglongbing (HLB). Breeding for disease‐resistant varieties is the most efficient and sustainable approach to control plant diseases. Traditional breeding of citrus varieties is challenging due to multiple limitations, including polyploidy, polyembryony, extended juvenility and long crossing cycles. Targeted genome editing technology has the potential to shorten varietal development for some traits, including disease resistance. Here, we used CRISPR/Cas9/sgRNA technology to modify the canker susceptibility gene *CsLOB1* in Duncan grapefruit. Six independent lines, D_LOB_2, D_LOB_3, D_LOB_9, D_LOB_10, D_LOB_11 and D_LOB_12, were generated. Targeted next‐generation sequencing of the six lines showed the mutation rate was 31.58%, 23.80%, 89.36%, 88.79%, 46.91% and 51.12% for D_LOB_2, D_LOB_3, D_LOB_9, D_LOB_10, D_LOB_11 and D_LOB_12, respectively, of the cells in each line. D_LOB_2 and D_LOB_3 showed canker symptoms similar to wild‐type grapefruit, when inoculated with the pathogen *Xanthomonas citri* subsp. citri (Xcc). No canker symptoms were observed on D_LOB_9, D_LOB_10, D_LOB_11 and D_LOB_12 at 4 days postinoculation (DPI) with Xcc. Pustules caused by Xcc were observed on D_LOB_9, D_LOB_10, D_LOB_11 and D_LOB_12 in later stages, which were much reduced compared to that on wild‐type grapefruit. The pustules on D_LOB_9 and D_LOB_10 did not develop into typical canker symptoms. No side effects and off‐target mutations were detected in the mutated plants. This study indicates that genome editing using CRISPR technology will provide a promising pathway to generate disease‐resistant citrus varieties.

## Introduction

Citrus varieties are high value tree crops with plantings in over one hundred countries. The fruit provides numerous benefits to human, including providing vitamins, fibre, calcium, potassium, folate and lowering health risks. Citrus production faces many biotic and abiotic challenges. Among them, the bacterial pathogens *Xanthomonas citri* ssp. citri (Xcc) and *Candidatus* Liberibacter asiaticus are the causal agents for citrus canker and HLB disease, respectively. Breeding disease‐resistant varieties is the most efficient and sustainable approach to control plant diseases. However, traditional citrus breeding has often been hindered by polyembryony, pollen‐ovule sterility, sexual and graft incompatibilities, and extended juvenility (Davey *et al*., 2005). Various biotechnology methods have been used to develop modified and novel citrus varieties (Chen *et al*., 2013; Dutt *et al*., 2015; Fu *et al*., 2011). However, no genetically modified varieties have been commercialized. The lack of commercial releases has been attributed to the lack of consumer acceptance of transgene technology. Recent developments in targeted genome editing technologies, however, have facilitated the process to establish genetically modified cultivars that lack transgenes in the final line (Doudna and Charpentier, 2014).

We previously identified *CsLOB1* as a critical citrus disease susceptibility gene for citrus canker (Hu *et al*., 2014). *CsLOB1* is a member of the Lateral Organ Boundaries Domain (LBD) gene family of plant transcription factors. All strains of Xcc and a related pathogen *X. fuscans* subsp. aurantifolii (Xfa) encode transcription activator‐like (TAL) effectors that recognize an effector binding element (EBE) in the promoter of *CsLOB1* and induce expression of the disease susceptibility gene (Hu *et al*., 2014). Furthermore, the EBEs of individual critical TAL effectors in various canker causing strains overlap (Hu *et al*., 2014). Thus, the EBE region of *CsLOB1* may be the Achilles’ heel of citrus canker and presents an attractive target for genomic engineering of broad resistance to citrus canker. Previously, genome modifications of EBE regions of susceptibility genes *Os11N3, Os14N3 and Os12N3* (also called *OsSWEET14, OsSWEET11* and *OsSWEET13*, respectively) have generated resistance in rice to bacterial blight, which is incited by *X. oryzae pv. oryzae* using a set of TAL effector genes related to the critical TAL effectors of Xcc and Xga (Blanvillain‐Baufumé *et al*., 2016; Li *et al*., 2012; Zhou *et al*., 2015). In our recent study, genome modification of the EBE of one single allele of *CsLOB1* gene in grapefruit Duncan (*Citrus paradisi* Macf.) alleviated the canker symptoms due to a specific TAL effector (Jia *et al*., 2016). However, the modified grapefruit line, which is a hybrid, is still susceptible to wild‐type Xcc as only one *CsLOB1* allele was altered, and mutation of the EBEs of both alleles of *CsLOB1* is required to generate reduced symptom plants (Jia *et al*., 2016). Here, we reported our progress to generate canker‐resistant citrus by disrupting the coding region of both alleles of *CsLOB1* using Cas9/sgRNA.

## Results

**Figure 1:**
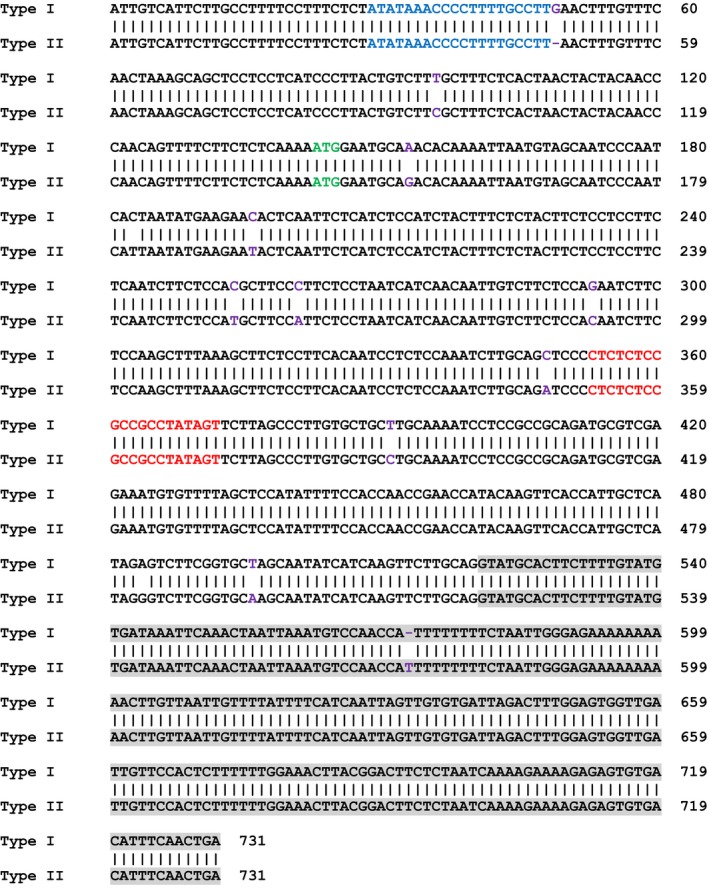
Alignment of Type I CsLOB1 and Type II CsLOB1 in Duncan grapefruit. Two alleles of *CsLOB1*, Type I and Type II, are present in Duncan grapefruit. Part of the promoter regions and coding sequences are shown, in which the difference was indicated by purple, and the PthA4 effector binding elements were highlighted by blue. The intron was highlighted in grey. The translation start site was highlighted in green. The sgRNA‐targeting region, which is conservative on both alleles, was highlighted in red. The primers were underlined, which were used to analyse indel mutation in genome‐modified Duncan by targeted next‐generation sequencing.

**Figure 2:**
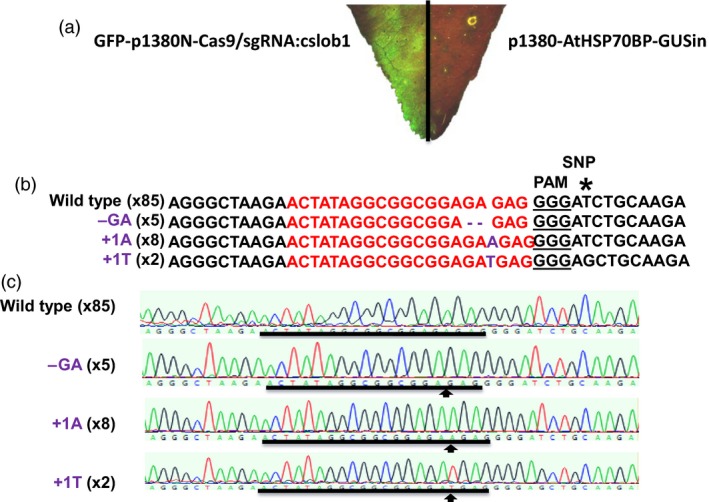
Function analysis of GFP‐p1380N‐Cas9/sgRNA:cslob1 in Duncan leaves with the aid of GFP. (a) Four days after agroinfiltration with *Agrobacterium* cells harbouring GFP‐p1380N‐Cas9/sgRNA:cslob1, GFP fluorescence was readily observed in Duncan grapefruit leaf. *Agrobacterium* cells harbouring p1380‐AtHSP70BP‐GUSin were used as a negative control. (b) GFP‐p1380N‐Cas9/sgRNA:cslob1‐directed modification to *CsLOB1* coding region. The GFP‐p1380N‐Cas9/sgRNA:cslob1‐targeted sequence in *CsLOB1* was shown in red, and the mutations were shown in purple. (c) The representative chromatograms of *CsLOB1* and its mutations. The targeted sequence within *CsLOB1* was underlined by black lines, and the mutant site was indicated with an arrow. Single nucleotide polymorphism (SNP) was indicated by an asterisk (*).

We first targeted the *CsLOB1* coding region using Cas9/sgRNA in a transient assay on Duncan grapefruit (*Citrus* ×* paradisi*), as grapefruit is one of the most canker susceptible citrus varieties. Grapefruit contains two alleles of *CsLOB1*, Type I and Type II (Jia *et al*., 2016) resulting from grapefruit being a hybrid of maternal donor pummelo (*C. maxima*) and paternal donor sweet orange (*C. sinensis*) (Velasco and Licciardello, 2014) (Figure 1). The two alleles of *CsLOB1* showed polymorphisms at both nucleotide and protein levels. The sgRNA was selected to target a conserved region of the 1st exon in both alleles (Figures 1 and S1). To facilitate the screen process, a binary vector GFP‐p1380N‐Cas9/sgRNA:cslob1, which contains a GFP reporter gene, was constructed (Figure S1). Citrus plants transformed with GFP‐p1380N‐Cas9/sgRNA:cslob could be readily monitored with GFP fluorescence. First, Xcc‐facilitated *Agrobacterium*‐mediated infiltration (Jia and Wang, 2014) and transient expression in citrus leaves were used to test GFP‐p1380N‐Cas9/sgRNA:cslob1 function. Four days after infiltration, GFP fluorescence was observed at the inoculation site, whereas no GFP signal was observed at the site of infiltration with the control vector p1380‐AtHSP70BP‐GUSin (Jia and Wang, 2014) (Figure 2a). PCR amplification and sequencing confirmed the targeted modification of *CsLOB1* (Figure 2b and c). Therefore, GFP‐p1380N‐Cas9/sgRNA:cslob1 is functional for *CsLOB1* coding region targeting.

**Figure 3:**
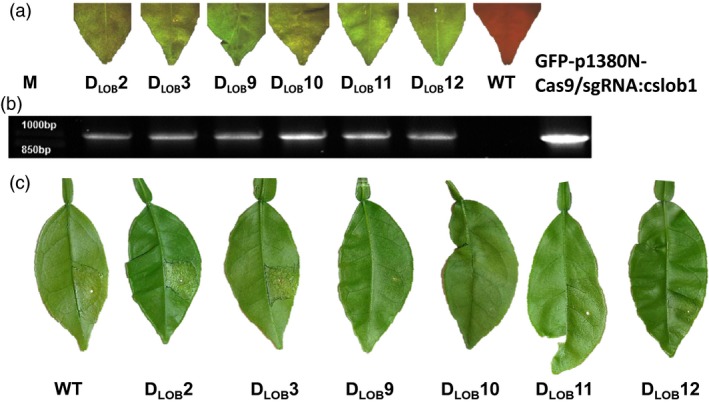
Analysis of GFP‐p1380N‐Cas9/sgRNA:cslob1‐transformed Duncan grapefruit. (a) six GFP‐p1380N‐Cas9/sgRNA:cslob1‐transformed Duncan grapefruit plants (D_LOB_2, D_LOB_3, D_LOB_9, D_LOB_10, D_LOB_11 and D_LOB_12) were GFP positive. The wild‐type grapefruit plant did not show GFP. (b) The six transgenic lines contain Cas9/sgRNA as indicated by PCR amplification using primers 35SP‐5‐P1 and NosP‐3‐P2. Plasmid GFP‐p1380N‐Cas9/sgRNA:cslob1 was used as a positive control. M, 1 kb DNA ladder; WT, wild type. C. The six *CsLOB1*‐modified lines showed differential resistance to Xcc. At 4 days postinoculation with Xcc (5 × 10^8^ CFU/mL) using needleless syringe, canker symptoms were observed on normal grapefruit, D_LOB_2 and D_LOB_3, but absent or reduced on D_LOB_9, D_LOB_10, D_LOB_11 and D_LOB_12.

**Figure 4:**
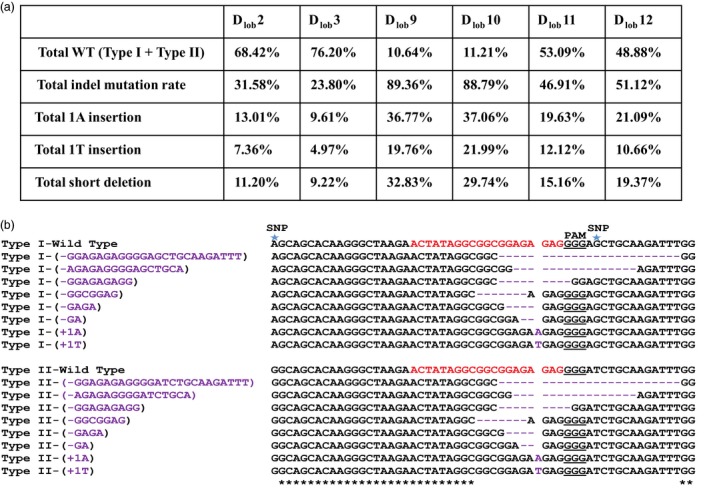
Indel mutation rates and mutation genotypes in six *CsLOB1*‐edited grapefruit lines. (a) Mutation rate for each *CsLOB1*‐edited grapefruit line. Targeted next‐generation sequencing was conducted for each line, and more than 50 000 paired‐end reads were generated for each sample. (b) Representative indel mutation genotypes in Type I *CsLOB1* plus Type II *CsLOB1*. The mutations included 1‐bp insertion and short deletions. It should be noted that the AGAGAGGGGA(G/T)CTGCA deletion and GGAGAGAGGGGA(G/T)CTGCAAGATTT deletion removed the PAM and the SNP nucleotide. Star indicates SNP (single nucleotide polymorphism) used for differentiating type I and type II alleles of *CsLOB1*.

Via *Agrobacterium*‐mediated transformation, Duncan grapefruit epicotyls were used as explants to create transgenic citrus plants (Orbović and Grosser, 2015). Six independent transgenic lines, D_LOB_2, D_LOB_3, D_LOB_9, D_LOB_10, D_LOB_11 and D_LOB_12, were selected based on GFP fluorescence and verified by PCR analyses (Figure 3a and b). To calculate the mutation frequency and determine the genotype for *CsLOB1* locus, targeted next‐generation sequencing of the six transgenic lines was performed on amplified fragments using primers targeting a 380‐bp region cover the targeted site. In total, more than 50 000 paired‐end reads were generated for each sample, and, after filtering and quality trimming, the reads were grouped to clusters with a threshold of 100% pairwise identity using UCLUST (Edgar, 2010) (Table S1). Based on the sequencing results, the mutation rate was 31.58%, 23.80%, 89.36%, 88.79%, 46.91% and 51.12% for D_LOB_2, D_LOB_3, D_LOB_9, D_LOB_10, D_LOB_11 and D_LOB_12, respectively (Figure 4a, Table S2). More than half of the mutations were 1‐bp insertions of A or T, resulting in frame shift (Figure 4b, Table S2). The majority of deletions were short, ranging from 2 bp to 22 bps. Most of the 2‐bp deletions were GA deletions (Figure 4b and Table S2). The 1‐bp insertions took place at the 4th bp upstream of the PAM site (Figure 4b). The GA and GAGA deletions also occurred three base pairs upstream of the PAM site (Figure 4b), which is consistent with the previous report that Cas9 nuclease cleaves target DNA at a position three base pairs upstream of the PAM sequence (Jinek *et al*., 2012). Lines D_LOB_9 and D_LOB_10 were further confirmed using Sanger sequencing analysis (Figure S2).

The susceptibility of the six *CsLOB1*‐modified Duncan grapefruit plants was tested by challenging with Xcc at the concentration of 5 × 10^8^ CFU/mL. D_LOB_2 and D_LOB_3 showed canker symptoms similar to wild‐type Duncan grapefruit. No canker symptoms were observed on D_LOB_9, D_LOB_10, D_LOB_11 and D_LOB_12 at 4 DPI (Figure 3c). Pustules caused by Xcc were observed on D_LOB_9, D_LOB_10, D_LOB_11 and D_LOB_12 in later stages, which were much reduced compared to that on wild‐type grapefruit. The pustules on D_LOB_9 and D_LOB_10 did not develop into typical canker symptoms, whereas reduced canker symptoms were observed on D_LOB_11 and D_LOB_12 (Figure S3). The appearance of the pustules on D_LOB_9 and D_LOB_10 might result from wild‐type cells or mutants that could not abolish the CsLOB1 function. We did not observe any visible phenotype change for *CsLOB1*‐edited grapefruit lines (Figure S4).

We analysed potential off‐target mutagenesis. Seven potential off‐target sequences were identified from genomic data (Table S3). No off‐target mutations in amplified fragments encompassing the sites were identified in the six *CsLOB1*‐modified plants (Table S3). Due to the somatic nature of the plants and only ten random colonies per putative off‐target site were sequenced, the possibility of off‐target changes in a fraction of the cells cannot be ruled out.

## Discussion

This study tested the proof of concept that we can generate canker‐resistant plants by modifying susceptibility gene *CsLOB1*. In this study, the mutation rate was 89.36% and 88.79% for D_LOB_9 and D_LOB_10, respectively, and both lines showed canker resistance (Figure 3c), even though pustules can be observed at later stage (Figure 3). The mutation rate for D_LOB_11, and D_LOB_12 was 46.91% and 51.12%, respectively. Both D_LOB_11 and D_LOB_12 showed enhanced resistance against Xcc with more pustules than D_LOB_9 and D_LOB_10 (Figures 3c, S3). On the other hand, the mutation rate for D_LOB_2 and D_LOB_3 was 31.58% and 23.80%, respectively. Neither D_LOB_2 nor D_LOB_3 showed resistance to citrus canker (Figures 3c, S3). The appearance of the pustules on D_LOB_9, D_LOB_10, D_LOB_11 and D_LOB_12 at 7 DPI might result from wild‐type cells or mutants that could not abolish the *CsLOB1* function. This recessive resistance due to mutation of *CsLOB1* is expected to be durable and efficient against all Xanthomonas pathotypes causing citrus canker because they all rely on induction of the susceptibility gene *CsLOB1* to induce canker symptoms. This is consistent with previous studies that mutation of the coding region of susceptibility gene will lead to disease resistance. Mutation of the susceptibility gene *OsSWEET13* corresponding to PthXo2 of *X. oryzae* pv. oryzae has generated disease‐resistant rice (Zhou *et al*., 2015). We need to point out that the genome‐modified citrus lines are not suitable for application to control citrus canker at this moment as they still contain Cas9 and sgRNA in the genome, thus are considered as transgenic, and require rigorous registration process before allowed for commercialization. Recently, USDA has granted nonregulatory status for the genome modified common white button mushroom (*Agaricus bisporus*) resisting against browning (Waltz, 2016b) and high amylopectin corn generated by knocking out the endogenous waxy gene Wx1 (Waltz, 2016a) because both do not contain foreign DNAs and are without off‐target mutations. Consequently, we need to generate nontransgenic canker‐resistant citrus varieties to facilitate the de‐regulation.

No phenotypic changes were observed for the *CsLOB1*‐modified plants. CsLOB1 belongs to the LBD proteins which are transcription factors in the regulation of plant growth and development (Husbands *et al*., 2007). The biological function of CsLOB1 remains to be determined. RNA‐Seq analysis of the expression profiles associated with CsLOB1 identified many downstream genes, for example cell organization, cell division, cell cycle, cell wall degradation and cell wall modification (Zhang *et al*., 2016). *PtaLBD1* derived from populus is a homolog of *CsLOB1*. It was reported that *PtaLBD1* was involved in secondary woody growth in poplar (Yordanov *et al*., 2010). The expression of *PtaLBD1‐SRDX*, harnessed for dominant‐negative suppression of PtaLBD1, suppressed stem diameter growth. No phenotypic changes observed for the *CsLOB1‐*modified plants are probably due to the fact that citrus contains multiple LOB genes with similar functions, for example *CsLOB1*,* CsLOB2* and *CsLOB3*. Induction of *CsLOB2* and *CsLOB3* using custom‐designed TAL effectors leads to similar canker symptoms due to induction of *CsLOB1* by PthA4 (Zhang *et al*., 2016), indicating similar functions of *CsLOB1*,* CsLOB2* and *CsLOB3*. Thus, redundancy of *CsLOB1* might be the main reason for the lack of phenotypic effect of the mutation. No off‐target mutations were observed in the genome‐modified plants, which might also contribute to the lack of phenotypic effect of the mutation. We could not totally rule out other phenotypic effects, for example flowering, as the genome‐modified lines will take 2 to 3 more years to flower.

In summary, we have shown that mutation of the coding region of both alleles of the susceptibility gene *CsLOB1* can generate citrus canker‐resistant plants. Future work needs to focus on generating *CsLOB1*‐modified citrus varieties which do not contain foreign DNA and comprise no off‐target mutations for application purpose. Importantly, this study showed that we can generate disease‐resistant citrus varieties using CRISPR technology and provide a long‐term and efficient control measurement for other citrus diseases including HLB.

## Materials and methods

### Plasmid construction

The CaMV 35S promoter was amplified using primers CaMV35‐5‐*Xho*I (5′‐ACTCGAGACTAGTACCATGGTGGACTCCTCTTAA‐3′) and sgRNA‐cslob1‐P1 (5′‐phosphorylated‐TATAGTCCTCTCCAAATGAAATGAACTTC‐3′), and the sgRNA‐NosT fragment was amplified using primers sgRNA‐cslob1‐P2 (5′‐phosphorylated‐ GGCGGCGGAGAGAGGTTTTAGAGCTAGAAATAGCAA‐3′) and NosT‐3‐*Asc*I (5′‐ACCTGGGCCCGGCGCGCCGATCTAGTAACATAGATGA‐3′). Through three‐way ligation, *Xho*I‐digested CaMV35S and *Asc*I‐cut sgRNA‐NosT were inserted into *Xho*I‐*Asc*I‐treated p1380N‐Cas9 to form p1380N‐Cas9/sgRNA:cslob1. The p1380N‐Cas9 was described previously (Jia and Wang, 2014).

Using a pair of primers 35T‐P1 (5′‐AGGTGGATCCGAGCTCGAAAATTTCTCCATAATAAT.

GTGTGAGT ‐3′) and 35T‐P2 (5′‐AGGTATTAATAAGCTTCGGGGGATCTGGATTTTAGTA CT‐3′), the CaMV 35S terminator was amplified and cloned into *Bam*HI‐*Ase*I‐digested p1380N‐Cas9 to produce p1380‐35S‐35T. The cassava vein mosaic virus promoter (CsVMV) was amplified using primers CsVMV‐5‐*Spe*I (5′‐AGGTACTAGTAAGCTTGCATGCCCGCGCC AGAAGGTAATTATCCAAG‐3′) and CsVMV‐3‐*Sal*I (5′‐AGGTGTCGACAAACTTACAAA TTTCTCTGAAG‐3′) from plasmid AtSUC2‐NPR1 (Dutt *et al*., 2015), and the GFP fragment was amplified using primers GFP‐5‐*Xho*I (5′‐AGGTCTCGAGATGAAGACTAATCTTT TTCTCT‐3′) and GFP2 (5′‐TCGAGCTCTTAAAGCTCATCATGTTTGTAT‐3′) from p1380‐35S‐GFP (Jia and Wang, 2014). Through three‐way ligation, *Spe*I‐CsVMV‐*Sal*I fragment and *Xho*I‐GFP‐*Sac*I were inserted into *Spe*I‐*Sac*I‐treated p1380‐35S‐35T to form p1380‐CsVMV‐GFP‐35T. After digestion with *Hin*dIII, the *Hin*dIII‐CsVMV‐GFP‐35T‐*Hin*dIII fragment from p1380‐CsVMV‐GFP‐35T was cloned into p1380N‐Cas9/sgRNA:cslob1 to obtain GFP‐p1380N‐Cas9/sgRNA:cslob1.

The binary vector GFP‐p1380N‐Cas9/sgRNA:cslob1 was introduced into *A. tumefaciens* strain EHA105 competent cells by the freeze–thaw method. Recombinant *Agrobacterium* cells were employed for citrus transformation or Xcc‐facilitated agroinfiltration.

### Duncan *CsLOB1* sequencing and analysis

Using a Wizard Genomic DNA Purification Kit (Promega), genomic DNA was extracted from wild‐type Duncan, or transgenic plants, or the GFP‐positive Duncan leaves treated by *Xanthomonas citri* ssp. citri (Xcc)‐facilitated agroinfiltration of GFP‐p1380N‐Cas9/sgRNA:cslob1 (Figure 3a). To analyse *CsLOB1* gene in detail, PCR was performed with the Phusion DNA polymerase (New England Biolabs) and a pair of primers, CsLBDP‐5‐P1 (5′‐ATTGTCATTCTTGCCTTTTCCTTTCT‐3′) and CsLOB1‐3‐P2 (5′‐TCAGTTGAAATGTCACACTCTCTT‐3′), flanking part of *CsLOB1* promoter and its coding region. By blunt end cloning, the PCR products were inserted into the PCR‐BluntII‐TOPO vector (Life Technologies). The colonies were randomly selected for DNA sequencing, and the results were visualized by Chromas Lite program.

For PCR product direct sequencing, CsLBDP‐5‐P1 and CsLOB1‐3‐P2 were used to amplify the DNA fragments from genomic DNA. The PCR products were purified and subjected to direct sequencing using primer CsLOB1‐P2 (5′‐TGAGCAATGGTGAACTTGTATGGTTC‐3′). The results were analysed by Chromas Lite program.

### Xcc‐facilitated agroinfiltration in Duncan grapefruit

Duncan grapefruit (*Citrus paradisi*) was grown in a glasshouse at temperatures ranging from 25 to 30 °C. Before Xcc‐facilitated agroinfiltration was carried out, the plants were pruned for uniform shoot establishment.

The detailed protocol for Xcc‐facilitated agroinfiltration in citrus leaves was described previously (Jia and Wang, 2014), with minor modification. Briefly, Duncan leaves were inoculated with a culture of actively growing XccΔgumC re‐suspended in sterile tap water (5 × 10^8^ CFU/mL). Twenty‐four hours later, the XccΔgumC‐treated leaf areas were agroinfiltrated with recombinant *Agrobacterium* cells harbouring GFP‐p1380N‐Cas9/sgRNA:cslob1 or p1380‐AtHSP70BP‐GUSin (Jia and Wang, 2014). Four days after agroinfiltration, leaves were subjected to GFP observation or genomic DNA extraction.

### GFP detection

Four days after Xcc‐facilitated agroinfiltration with GFP‐p1380N‐Cas9/sgRNA:cslob1 or p1380‐AtHSP70BP‐GUSin, GFP fluorescence in the treated leaves was visualized under illumination of an EBQ 100 isolated light source using a Zeiss Stemi SV11 dissecting microscope equipped with an Omax camera. The leaf was photographed using the Omax Toupview software.

### *Agrobacterium*‐mediated Duncan grapefruit transformation

Citrus transformation was performed as reported before (Orbović and Grosser, 2015). In detail, about 2923 Duncan epicotyl explants were co‐incubated with recombinant *Agrobacterium* cells harbouring binary vector GFP‐p1380N‐Cas9/sgRNA:cslob1. Five weeks later, about 839 shoots sprouted from these explants after co‐incubation. All explants were inspected for the presence of GFP fluorescence. In the initial screen, 15 shoots were designated as positive and micro‐grafted on ‘Carrizo’ citrange rootstock plants [*Citrus sinensis* (L.) Osbeck × *Poncirus trifoliata* (L.) Raf.]. Out of these shoots, seven died upon grafting in *in vitro* conditions before they were transferred to pots. Additional two plants were discarded based on unsatisfactory level of GFP fluorescence detected in their tissue during secondary inspection. The six remaining GFP‐positive plants were used for further analysis.

The GFP‐p1380N‐Cas9/sgRNA:cslob1‐transformed plants were subjected to PCR analysis with a pair of primers, 35SP‐5‐P1 (5′‐ATCAAAGGCCATGGAGTCAAA‐3′) and NosP‐3‐P2 (5′‐TTGTCGTTTCCCGCCTTCAGT‐3′).

### Next‐generation sequencing analysis

Genomic DNA from six transgenic plants was used as template for PCR amplification using a pair of primers, CsLOB1‐P1 (5′‐TCTCACTAACTACTACAACCCAACAG‐3′) and CsLOB1‐P2 (Figure 1). All PCR products were pooled to construct the DNA library for sequencing using an Illumina HiSeq 2500 platform at Novogene (Beijing, China). For each sample, more than 50 000 paired‐end reads were generated. After de‐multiplex, barcode and primer deletion using custom Perl script, the raw reads were quality trimmed using sickle software with parameters average quality 30 and reads length threshold 200 bp (Fass *et al*., 2011). The remaining high‐quality reads were clustered with a threshold of 100% pairwise identity using UCLUST (Edgar, 2010). The representative sequences from abundant clusters with relative abundance >1% were aligned using MEGA 6 (Tamura *et al*., 2013) and further analysed for mutation genotype.

### Xcc infection assay

Wild‐type Duncan grapefruit and *CsLOB1*‐modified grapefruit lines were grown in a glasshouse. The same age leaves were inoculated with Xcc (5 × 10^8^ CFU/mL) using a needleless syringe. After inoculation, citrus canker formation was observed and photographed at different time points.

### Analysis of potential off‐targets

To analyse potential off‐targets of GFP‐p1380N‐Cas9/sgRNA:cslob1 in *CsLOB1*‐modified grapefruit lines, we analysed the putative off‐targets using a web‐based software (http://cbi.hzau.edu.cn/cgi-bin/CRISPR)**.** Genomic DNA from *CsLOB1*‐modified grapefruit lines was used as template, and the primers listed in Table S3 were used to amplify the fragment containing the off‐targets. Finally, the PCR products were ligated with PCR‐BluntII‐TOPO vector for sequencing analysis.

## Supporting information


**Figure S1**. Schematic diagram of GFP‐p1380N‐Cas9/sgRNA:cslob1. CaMV 35S and 35T, the cauliflower mosaic virus 35S promoter and its terminator; NosP and NosT, the nopaline synthase gene promoter and its terminator; LB and RB, the left and right borders of the T‐DNA region; Flag‐Cas9‐NLS, the Cas9 endonuclease containing Flag tag at its N‐terminal and nuclear location signal at its C‐terminal; target, the 20 nucleotides of *CsLOB1* highlighted by red, was conserved on both alleles; sgRNA scaffold, a synthetic single‐guide RNA composed of a fusion of CRISPR RNA and *trans*‐activating CRISPR RNA; NptII, neomycin phosphotransferase II; GFP, green fluorescent protein; CsVMV, the cassava vein mosaic virus promoter; PAM, protospacer‐adjacent motif.
**Figure S2.** Representative chromatograms of *CsLOB1* and its mutations in D_LOB_9 and D_LOB_10. Representative chromatograms of *CsLOB1* and its mutations in D_LOB_9 transgenic plant (a, b) and #D_LOB_10 transgenic plant (c, d). The targeted sequence within *CsLOB1* was shown by black lines, and the mutant site was pointed out by an arrow. Star indicates SNP.
**Figure S3.** The six Duncan transgenic lines showing differential resistance to Xcc. At 7 days postinoculation with Xcc (5 × 10^8 ^CFU/mL), severe canker symptoms were observed on wild type grapefruit, D_LOB_2 and D_LOB_3. Reduced canker symptoms were present on D_LOB_9, D_LOB_10, D_LOB_11 and D_LOB_12.
**Figure S4.** No visible phenotypic changes for GFP‐p1380N‐Cas9/sgRNA:cslob1‐transformed Duncan grapefruit lines. The GFP‐p1380N‐Cas9/sgRNA:cslob1‐transformed plants were grown in glasshouse.

**Table S1:** Overview of the next generation sequencing data.

**Table S2:** Analysis of indel mutations for Type I *CsLOB1* and Type II *CsLOB1* in six transgenic Duncan grapefruit lines.

**Table S3:** Potential off‐targets in transgenic Duncan grapefruit.
