# Supplementary material for: Genome editing of the disease susceptibility gene CsLOB1 in citrus confers resistance to citrus canker
Source: Plant Biotechnol J. 2017 Jan 4;15(7):817–23. doi: 10.1111/pbi.12677 (PMC5466436; doi:10.1111/pbi.12677)
Supplement: Supplementary file 1 — Figure S1. Schematic diagram of GFP‐p1380N‐Cas9/sgRNA:cslob1. CaMV 35S and 35T, the cauliflower mosaic virus 35S promoter and its terminator; NosP and NosT, the nopaline synthase gene promoter and its terminator; LB and RB, the left and right borders of the T‐DNA region; Flag‐Cas9‐NLS, the Cas9 endonuclease containing Flag tag at its N‐terminal and nuclear location signal at its C‐terminal; target, the 20 nucleotides of CsLOB1 highlighted by red, was conserved on both alleles; sgRNA scaffold, a synthetic single‐guide RNA composed of a fusion of CRISPR RNA and trans‐activating CRISPR RNA; NptII, neomycin phosphotransferase II; GFP, green fluorescent protein; CsVMV, the cassava vein mosaic virus promoter; PAM, protospacer‐adjacent motif. Figure S2. Representative chromatograms of CsLOB1 and its mutations in DLOB9 and DLOB10. Representative chromatograms of CsLOB1 and its mutations in DLOB9 transgenic plant (a, b) and #DLOB10 transgenic plant (c, d). The targeted sequence within CsLOB1 was shown by black lines, and the mutant site was pointed out by an arrow. Star indicates SNP. Figure S3. The six Duncan transgenic lines showing differential resistance to Xcc. At 7 days postinoculation with Xcc (5 × 10^8^ CFU/mL), severe canker symptoms were observed on wild type grapefruit, DLOB2 and DLOB3. Reduced canker symptoms were present on DLOB9, DLOB10, DLOB11 and DLOB12. Figure S4. No visible phenotypic changes for GFP‐p1380N‐Cas9/sgRNA:cslob1‐transformed Duncan grapefruit lines. The GFP‐p1380N‐Cas9/sgRNA:cslob1‐transformed plants were grown in glasshouse. [file PBI-15-817-s003.pptx]

## Slide 1
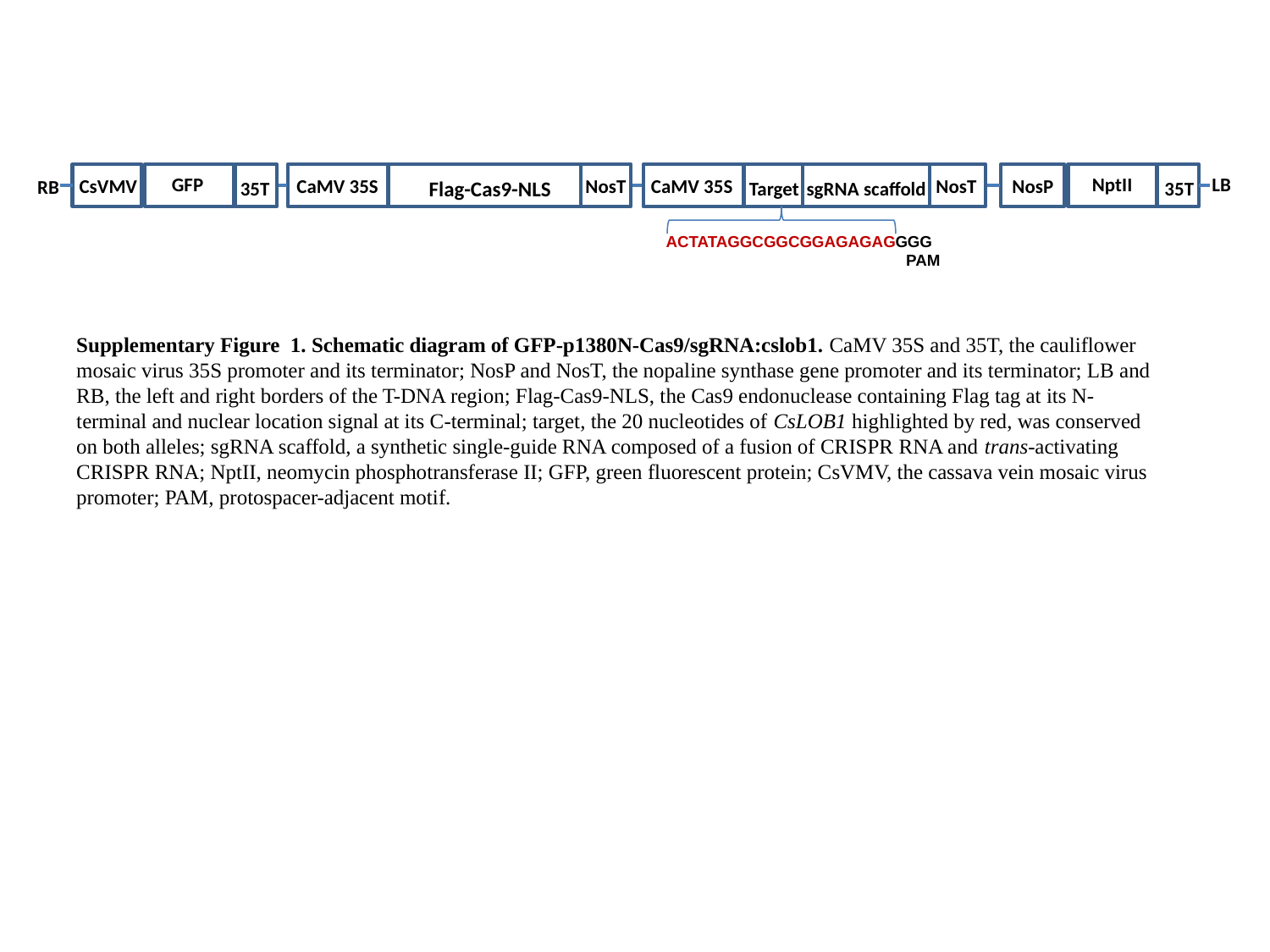

GFP
NptII
CsVMV
CaMV 35S
NosT
CaMV 35S
NosT
NosP
RB
35T
Flag-Cas9-NLS
Target
sgRNA scaffold
35T
 ACTATAGGCGGCGGAGAGAGGGG
 PAM
LB
Supplementary Figure 1. Schematic diagram of GFP-p1380N-Cas9/sgRNA:cslob1. CaMV 35S and 35T, the cauliflower mosaic virus 35S promoter and its terminator; NosP and NosT, the nopaline synthase gene promoter and its terminator; LB and RB, the left and right borders of the T-DNA region; Flag-Cas9-NLS, the Cas9 endonuclease containing Flag tag at its N-terminal and nuclear location signal at its C-terminal; target, the 20 nucleotides of CsLOB1 highlighted by red, was conserved on both alleles; sgRNA scaffold, a synthetic single-guide RNA composed of a fusion of CRISPR RNA and trans-activating CRISPR RNA; NptII, neomycin phosphotransferase II; GFP, green fluorescent protein; CsVMV, the cassava vein mosaic virus promoter; PAM, protospacer-adjacent motif.

## Slide 2
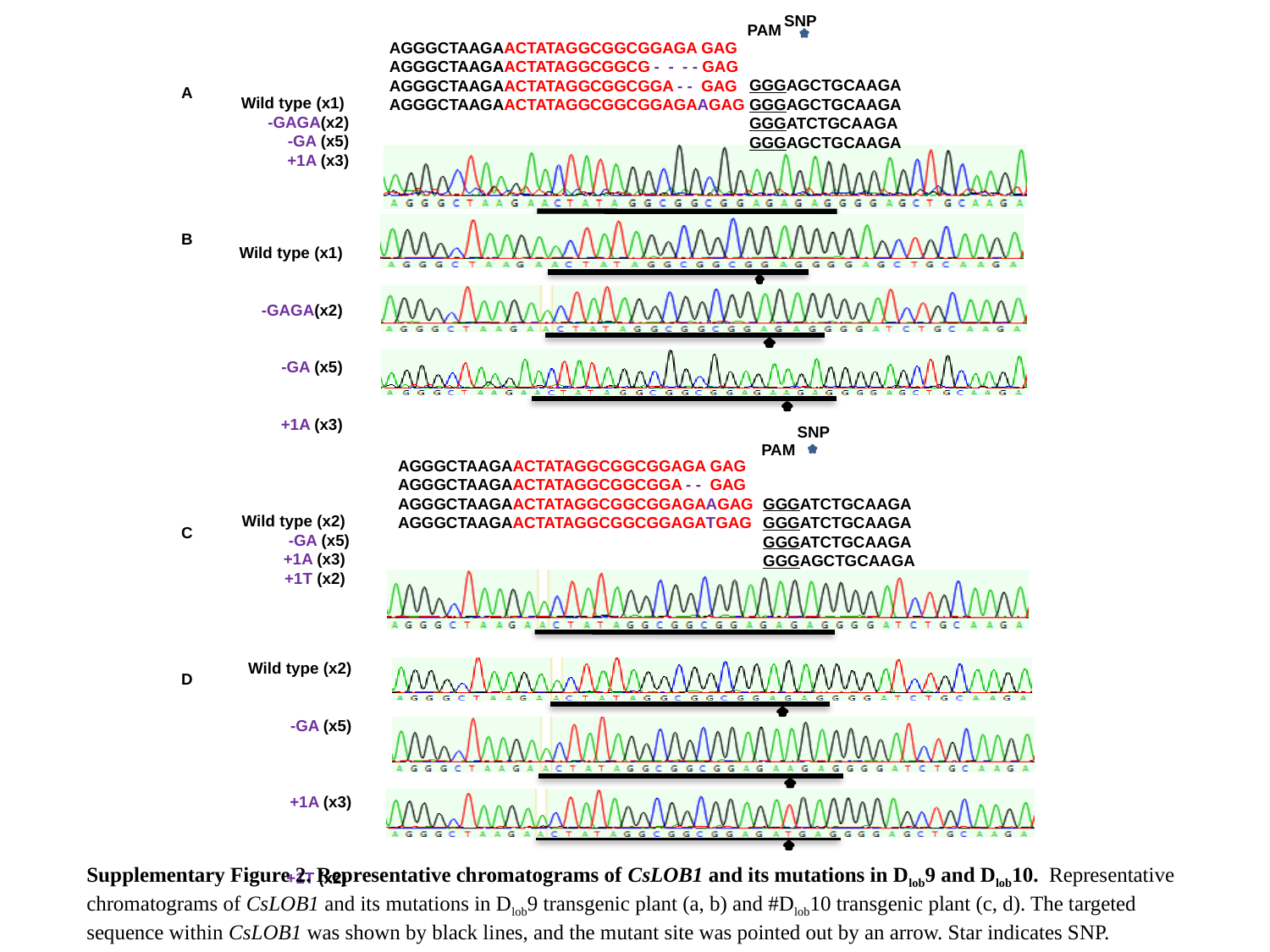

Wild type (x1)
-GAGA(x2)
-GA (x5)
 +1A (x3)
GGGAGCTGCAAGA
GGGAGCTGCAAGA
GGGATCTGCAAGA
GGGAGCTGCAAGA
AGGGCTAAGAACTATAGGCGGCGGAGA GAG
AGGGCTAAGAACTATAGGCGGCG - - - - GAG
AGGGCTAAGAACTATAGGCGGCGGA - - GAG
AGGGCTAAGAACTATAGGCGGCGGAGAAGAG
SNP
PAM
Wild type (x1)
-GAGA(x2)
-GA (x5)
+1A (x3)
A
B
C
D
Wild type (x2)
-GA (x5)
+1A (x3)
+1T (x2)
GGGATCTGCAAGA
GGGATCTGCAAGA
GGGATCTGCAAGA
GGGAGCTGCAAGA
AGGGCTAAGAACTATAGGCGGCGGAGA GAG
AGGGCTAAGAACTATAGGCGGCGGA - - GAG
AGGGCTAAGAACTATAGGCGGCGGAGAAGAG
AGGGCTAAGAACTATAGGCGGCGGAGATGAG
PAM
SNP
Wild type (x2)
-GA (x5)
 +1A (x3)
+1T (x2)
Supplementary Figure 2. Representative chromatograms of CsLOB1 and its mutations in Dlob9 and Dlob10. Representative chromatograms of CsLOB1 and its mutations in Dlob9 transgenic plant (a, b) and #Dlob10 transgenic plant (c, d). The targeted sequence within CsLOB1 was shown by black lines, and the mutant site was pointed out by an arrow. Star indicates SNP.

## Slide 3
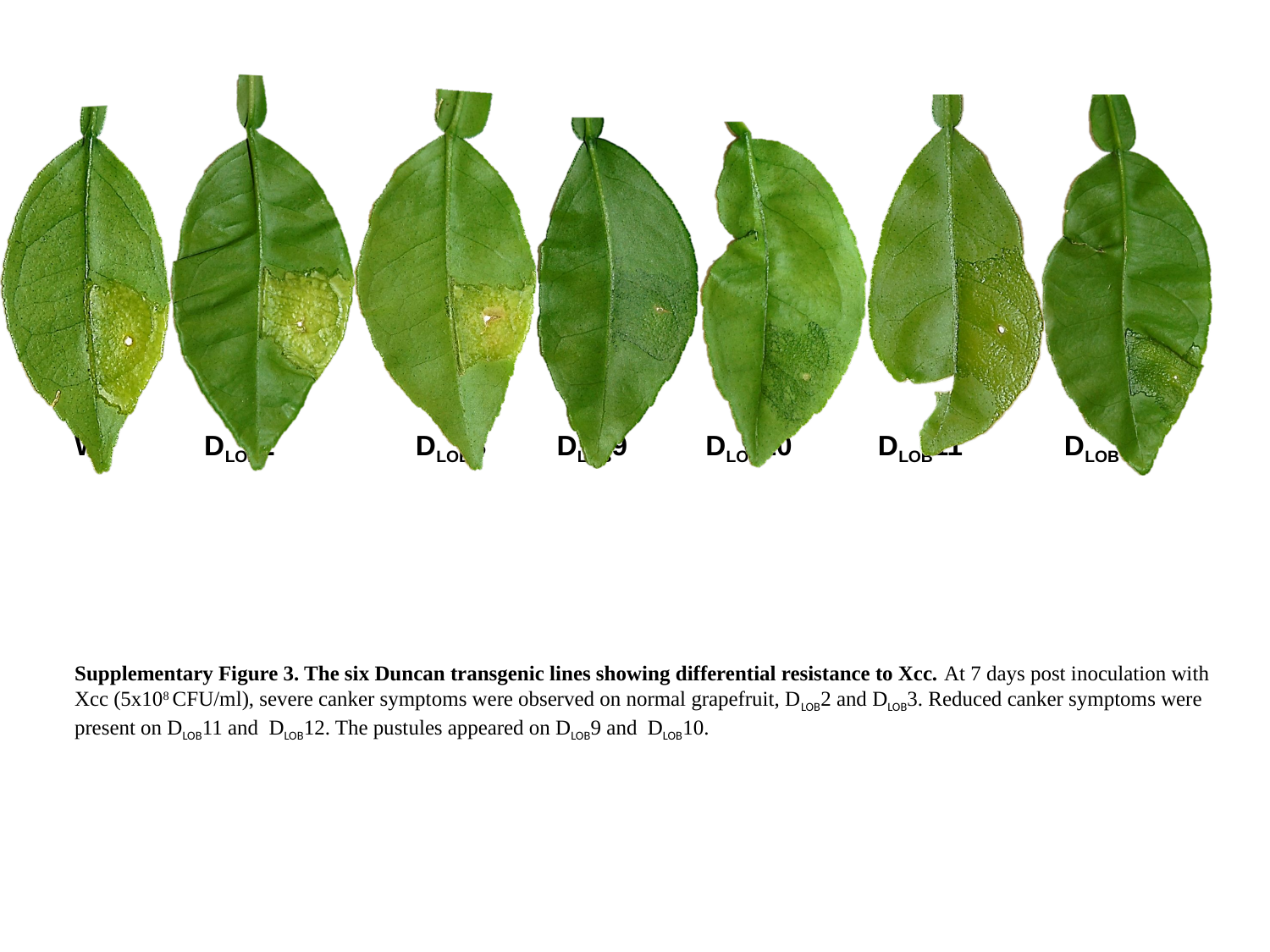

WT DLOB2 DLOB3 DLOB9 DLOB10 DLOB11 DLOB12
Supplementary Figure 3. The six Duncan transgenic lines showing differential resistance to Xcc. At 7 days post inoculation with Xcc (5x10^8^ CFU/ml), severe canker symptoms were observed on normal grapefruit, DLOB2 and DLOB3. Reduced canker symptoms were present on DLOB11 and DLOB12. The pustules appeared on DLOB9 and DLOB10.

## Slide 4
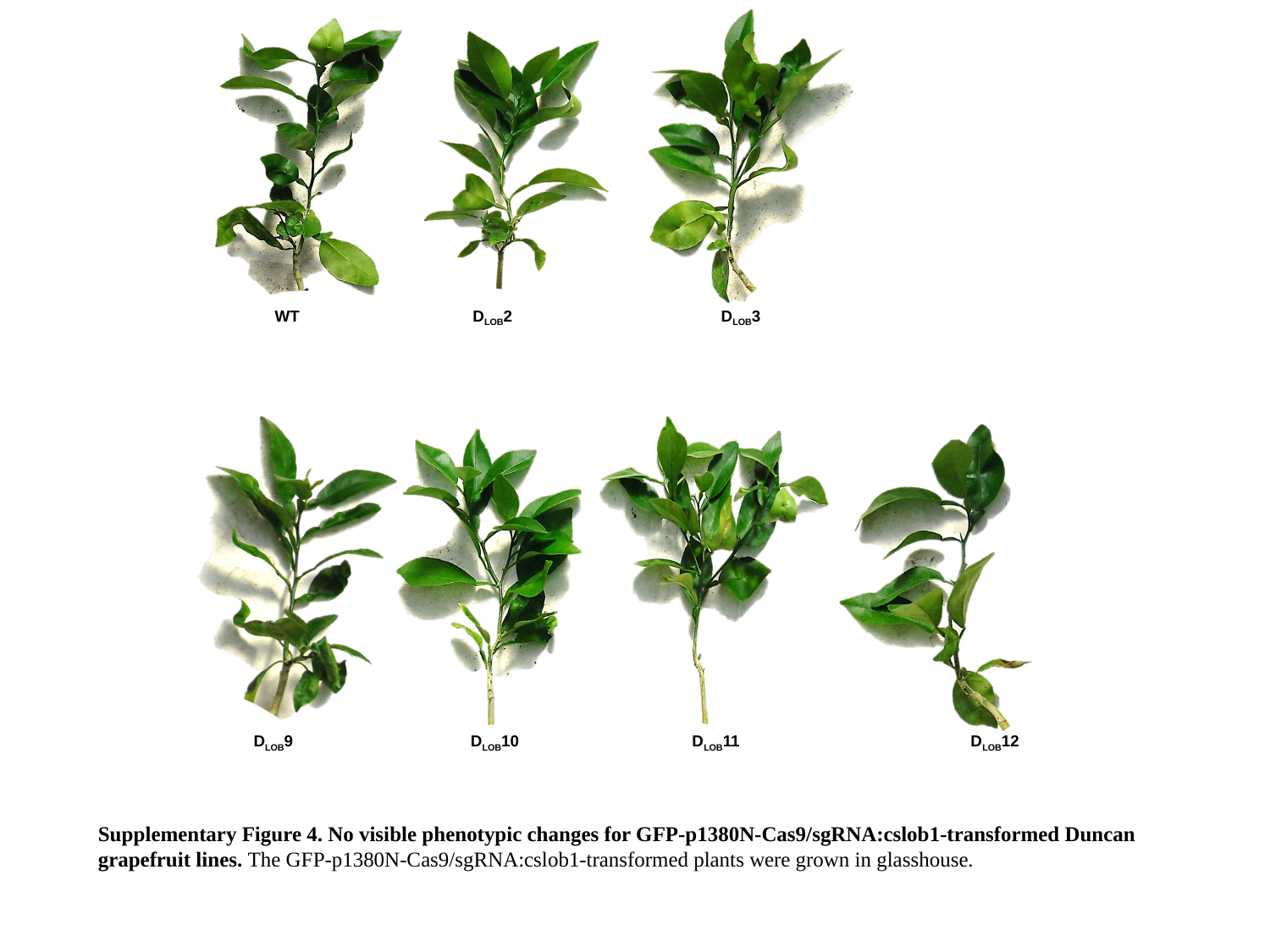

WT DLOB2 DLOB3
DLOB9 DLOB10 DLOB11 DLOB12
Supplementary Figure 4. No visible phenotypic changes for GFP-p1380N-Cas9/sgRNA:cslob1-transformed Duncan grapefruit lines. The GFP-p1380N-Cas9/sgRNA:cslob1-transformed plants were grown in glasshouse.
