# Supplementary material for: Genome editing of the disease susceptibility gene CsLOB1 in citrus confers resistance to citrus canker
Source: Plant Biotechnol J. 2017 Jan 4;15(7):817–23. doi: 10.1111/pbi.12677 (PMC5466436; doi:10.1111/pbi.12677)
Supplement: Supplementary file 2 — Table S1: Overview of the next generation sequencing data. [file PBI-15-817-s002.docx]

**Supplementary Table 1: Overview of the next generation sequencing data**

| **Sample Name** | **No. raw reads** | **No. high quality reads** |
| --- | --- | --- |
| D_LOB_2 | 52506 | 11973 |
| D_LOB_3 | 58019 | 11638 |
| D_LOB_9 | 68551 | 11882 |
| D_LOB_10 | 52677 | 9718 |
| D_LOB_11 | 53699 | 10816 |
| D_LOB_12 | 58335 | 8201 |

Note: Since this is an amplicon-based analysis, the reads fully covered the target region. The target coverage is equal to the No. high quality reads.
