# Supplementary material for: Genome editing of the disease susceptibility gene CsLOB1 in citrus confers resistance to citrus canker
Source: Plant Biotechnol J. 2017 Jan 4;15(7):817–23. doi: 10.1111/pbi.12677 (PMC5466436; doi:10.1111/pbi.12677)
Supplement: Supplementary file 3 — Table S2: Analysis of indel mutations for Type I CsLOB1 and Type II CsLOB1 in six transgenic Duncan grapefruit lines. [file PBI-15-817-s004.docx]

**Supplementary Table 2: Analysis of indel mutations for Type I *CsLOB1*and Type II *CsLOB1*in six transgenic Duncan grapefruit lines**

|  | #D_LOB_2 | #D_LOB_3 | #D_LOB_9 | #D_LOB_10 | #D_LOB_11 | #D_LOB_12 |
| --- | --- | --- | --- | --- | --- | --- |
| Type I-Wild Type | 28.20% | 45.45% | 3.08% | 3.77% | 23.35% | 16.00% |
| Type I-(-GGAGAGAGGGGAGCTGCAAGATTT) | 0.82% | 0.26% | 1.75% | 1.86% | 0.44% | 1.11% |
| Type I-(-AGAGAGGGGAGCTGCA) | 0.37% | 0.25% | 0.60% | 0.46% | 0.33% | 0.60% |
| Type I-(-GGAGAGAGG) | 0.47% | 0.14% | 0.65% | 0.51% | 0.28% | 0.44% |
| Type I-(-GGCGGAG) | 0.37% | 0.40% | 0.77% | 0.62% | 0.47% | 0.47% |
| Type I-(-GAGA) | 0.47% | 0.39% | 1.32% | 1.11% | 0.59% | 0.98% |
| Type I-(-GA) | 3.20% | 2.95% | 8.75% | 7.69% | 4.85% | 6.34% |
| Type I-(+1A) | 7.96% | 5.64% | 18.52% | 18.72% | 11.05% | 12.31% |
| Type I-(+1T) | 4.85% | 3.20% | 10.44% | 12.10% | 7.05% | 6.08% |
|  |  |  |  |  |  |  |
| Type II-Wild Type | 40.22% | 30.75% | 7.56% | 7.44% | 29.74% | 32.88% |
| Type II-(-GGAGAGAGGGGATCTGCAAGATTT) | 0.79% | 0.30% | 1.71% | 1.83% | 0.69% | 1.07% |
| Type II-(-AGAGAGGGGATCTGCA) | 0.23% | 0.14% | 0.62% | 0.70% | 0.21% | 0.42% |
| Type II-(-GGAGAGAGG) | 0.02% | 0.06% | 0.09% | 0.10% | 0.00% | 0.00% |
| Type II-(-GGCGGAG) | 0.44% | 0.70% | 1.28% | 1.15% | 0.58% | 0.51% |
| Type II-(-GAGA) | 0.49% | 0.45% | 1.92% | 1.93% | 0.69% | 1.16% |
| Type II-(-GA) | 3.53% | 3.19% | 13.37% | 11.78% | 6.01% | 6.27% |
| Type II-(+1A) | 5.05% | 3.97% | 18.25% | 18.34% | 8.58% | 8.78% |
| Type II-(+1T) | 2.51% | 1.77% | 9.32% | 9.89% | 5.08% | 4.58% |
|  |  |  |  |  |  |  |
| Total Type I insertion (1A+1T) | 12.81% | 8.84% | 28.96% | 30.82% | 18.10% | 18.39% |
| Total Type II insertion (1A+1T) | 7.56% | 5.74% | 27.57% | 28.23% | 13.65% | 13.36% |
| Total Type I short deletion | 5.70% | 4.38% | 13.83% | 12.26% | 6.96% | 9.94% |
| Total Type II short deletion | 5.51% | 4.84% | 18.99% | 17.48% | 8.20% | 9.43% |
