# Supplementary material for: Genome editing of the disease susceptibility gene CsLOB1 in citrus confers resistance to citrus canker
Source: Plant Biotechnol J. 2017 Jan 4;15(7):817–23. doi: 10.1111/pbi.12677 (PMC5466436; doi:10.1111/pbi.12677)
Supplement: Supplementary file 4 — Table S3: Potential off‐targets in transgenic Duncan grapefruit. [file PBI-15-817-s001.pptx]

## Slide 1
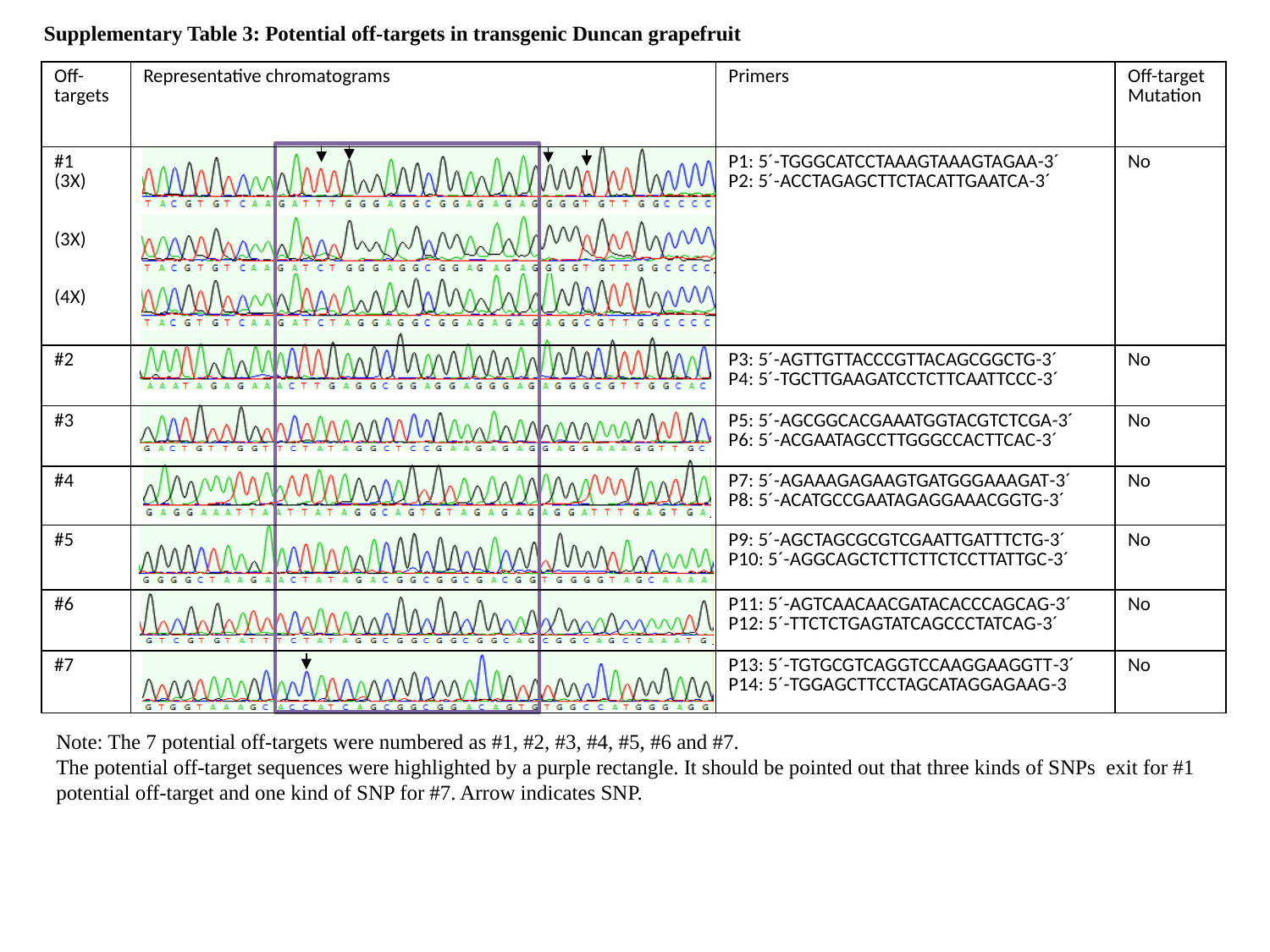

Supplementary Table 3: Potential off-targets in transgenic Duncan grapefruit
| Off-targets | Representative chromatograms | Primers | Off-target Mutation |
| --- | --- | --- | --- |
| #1 (3X) (3X) (4X) | | P1: 5´-tgggcatcctaaagtaaagtagaa-3´ P2: 5´-acctagagcttctacattgaatca-3´ | No |
| #2 | | P3: 5´-agttgttacccgttacagcggctg-3´ P4: 5´-tgcttgaagatcctcttcaattccc-3´ | No |
| #3 | | P5: 5´-agcggcacgaaatggtacgtctcga-3´ P6: 5´-acgaatagccttgggccacttcac-3´ | No |
| #4 | | P7: 5´-agaaagagaagtgatgggaaagat-3´ P8: 5´-acatgccgaatagaggaaacggtg-3´ | No |
| #5 | | P9: 5´-agctagcgcgtcgaattgatttctg-3´ P10: 5´-aggcagctcttcttctccttattgc-3´ | No |
| #6 | | P11: 5´-agtcaacaacgatacacccagcag-3´ P12: 5´-ttctctgagtatcagccctatcag-3´ | No |
| #7 | | P13: 5´-tgtgcgtcaggtccaaggaaggtt-3´ P14: 5´-tggagcttcctagcataggagaag-3 | No |
Note: The 7 potential off-targets were numbered as #1, #2, #3, #4, #5, #6 and #7.
The potential off-target sequences were highlighted by a purple rectangle. It should be pointed out that three kinds of SNPs exit for #1 potential off-target and one kind of SNP for #7. Arrow indicates SNP.
